# A Report on Fever

**Published:** 1847-04

**Authors:** David Prince

**Affiliations:** Jacksonville, Illinois


ILLINOIS AND INDIANA

MEDICAL AND SURGICAL JOURNAL.

Vol. II. NEW SERIES.—APRIL, 1847. No. 1.

### PART I.—ORIGINAL COMMUNICATIONS.

ARTICLE I.

*A Report on Fever.* Read before the Morgan County Medical Society, November 2, 1846: By DAVID PRINCE, M. D., of Jacksonville, Illinois.

The practitioner accustomed to see disease as manifested in the primitive and transitional geological regions of the north-eastern portion of the United States, will be struck with the comparative rareness, here, of inflammatory affections; and the student who gets his ideas of disease from English authors, and from those who reside along our northern Atlantic coast, will find a great discrepancy between the *book* and the *patient* when he comes to apply his reading to practice. The writer has seen, during the past nine years, but few cases of inflammatory fever, and they were colored to some extent with the malarial agency which prevails every where on our alluvial soil. During the writer's two years' experience in Morgan county, he has not observed any general difference in the severity of the fevers, or their tendency to local affections, though the number of cases, in this region, has seemed to be less the present year than during the preceding. In each of these years, a wet spring has been followed by a long, dry, and hot summer. Nor is the writer aware that any *great* change in the character of disease has occurred during the last nine years, though the fevers of 1837, 1838, 1839, and 1840, bore depleting treatment better, and manifested less tendency to prostration than those which have fallen under his observation since; and, as far as he has been able to learn the experience of other physicians, this increased tendency to a typhoid type, has been general throughout this and the adjoining States in the same latitude. This typhoid tendency, with our better knowledge of the therapeutic effects of Sulphate of Quinia, has led to the use of this remedy in doses and under circumstances which were not dreamed of ten years ago. No one now thinks of depending upon *mercurial action* to interrupt the progress of a remitting fever; but as soon as he has made, by the exhibition of an emetic, a cathartic, or an emeto-cathartic, an impression upon the glands whose secretion has been deranged, he endeavors, at once, to interrupt the paroxysms by the free use of Quinia, alone or in some combination; supposing that, if he does not succeed in arresting the fever, he will obviate that *sinking tendency* which has so often disappointed the hopes of both patient and physician.

Experience also proves that those local determinations so often witnessed in fever, in which the brain, the liver, the spleen, and sometimes the lungs, are made the passive recipients of a larger quantity of blood than they are wont to contain, are best removed by agents which promptly control the fever, and that any treatment of the local affection, unless accompanied by that which will cut short the fever, or tend to do it, will be ineffectual, and that as often as a remission or a chill followed by a febrile paroxysm is allowed to recur, there is great danger of an aggravation of the local plethora, and risk, also, of the formation of new congestions, disturbing the action of organs whose functions are essential to life. And if these congestions are passive—and passive they must be—to be removed by brandy, quinia, and opium, assisted, when the brain is affected, by cold, which probably acts as an indirect stimulant upon the blood-vessels—in what are they radically different from those general internal congestions which take place every time a chill occurs, and which, in the cases manifesting the greatest *sinking* tendency, are not entirely recovered from with the re-action which follows?

So much is this congestion like inflammation, while so little of its real nature does it possess, that the writer has witnessed a cerebral congestion, accompanied by all the distressing symptoms of phrenitis, subside in a few hours, leaving no trace of its existence behind, while, as the cerebral symptoms disappeared, others, equally resembling those of inflammation, occurred in the abdomen, and as the patient died previously to the subsidence of these, the autopsie exhibited a distinctly hyperematous appearance beneath the peritoneal, and the mucous surfaces, though the brain showed no mark of disease.

Though the different varieties of fever of malarial origin run into each other, so that if any standard of division is established, it is extremely difficult to fix the location of many examples which come before us, yet it will be convenient to divide them into four classes, which have again almost as many varieties as there are individual cases.

I. *Of Simple Intermittent Fever* it is not my design now to speak. Suffice it to say it seems to be owing to the same causes which produce remittents, or to causes similar though less intense; and the same treatment which cures the one is applicable, with modifications, to the other; and an intermittent is frequently a precursor of a remittent or a sequel to it.

II. *Remitting Fever of the Simple Form* begins, usually, with a chill, and is followed by an exacerbation of fever which subsides, and after a remission of the febrile symptoms, another exacerbation follows the next day without being preceded by any distinctly marked chill. Sometimes the remitting is preceded by an intermitting fever of three or four paroxysms, and then the chilly *usher* disappears and the fever acquires a more formidable character. These paroxysms recur daily, sometimes more aggravated every alternate day, until the fever subsides, in from seven to fourteen days, in death or convalescence.

III. *Typhoid Form.* In the third form of fever, the patient is usually unwell, for some time before the occurrence of either chill or fever; and when the patient is interrogated he will hardly confess that he has either a chill or a fever, but says they are "all mixed up together;" feeling cold creeping sensations at the same time that hot flashes pass in different directions, and frequently one sensation is quickly followed by the other. The friends or spectators frequently doubt whether there is any fever except, perhaps, an "*inward* fever," such is the absence of heat which is so often thought to be the essential idea of fever. The secretions, however, are arrested or perverted, the tongue is dry and coated, but not thickly, and soon becomes dark, and sometimes almost black; occasionally the coating cracks, moistens, peels off, and, in protracted cases, it is followed by a new coating.

Though the fever of this form has, as seen by the writer, been generally capable of control by appropriate and energetic treatment, it does not yield for several days, and, before subsiding, acquires almost a continued form, and so gradually does it let go its grasp, that it is difficult to decide when the fever ends or convalescence begins. When inappropriately treated, or left to itself, a typhoid tendency is manifest, favoring a fatal termination, and far more likely to do it when the disease is treated on a mercurial and depleting plan than when it is left entirely to its own tendencies. To this form physicians have sometimes given the name *Congestive*, seldom applying the term, however, till the occurrence of the latter stages of the affection, but *Typhoid Remittent* would certainly be a better appellation.

IV. *Congestive Form.* Congestive Fever applies far more appropriately to the course of symptoms now to be enumerated. The attack generally occurs without much premonition, and often suddenly, in circumstances of exposure to powerful malarious influence. Whether there is much chilliness or not, the prostration is very great from the beginning, and such a degree of stupor takes place, in the great majority of instances, as greatly to obscure the intellectual manifestations, or entirely to obstruct or conceal them. The heart is variously influenced, sometimes making a hundred and thirty beats in a minute, while, at other times, the heart's action seems unaltered or the pulsations are even fewer than natural, with an entire absence of febrile excitement. Whatever other changes in the pulse may be noticed, however, a strong pulse rarely or never occurs in this form of disease, for, though a considerable degree of fulness exist, the finger, placed firmly upon the artery, easily arrests the current.

Difficult and imperfect inspiration, with evident deficiency of the circulation at the surface, and sometimes a leaden hue of the skin, indicate a state of congestion of the internal organs and a want of power to send the blood, with its wonted energy and freedom, throughout the system. The remissions after, amount to a perfect intermission, when, in common language, we have, instead of *Congestive Fever* applied to the remitting form, the appellation of *Congestive Chill*, and some writers have, not without reason, proposed to use the simple word *Congestion* for these states when unaccompanied by evident fever. Of the remitting form of this variety, the writer has seen but few instances, and they were in the midst of summer, and in localities the most unhealthy, though, in more southern latitudes, this form of disease seems to be of frequent occurrence. The intermitting form, (*Congestive Chill*,) is not unfrequently seen in this climate and in this region, and is more rapidly fatal than the remitting, though less probably so if treated early and energetically. Without treatment, life is often terminated in the second or third paroxysm, and even sometimes in the first. The treatment of these diseases is, at present, far from being uniform, though there seems, now, to be an approximation to a more effectual plan than any with which our *classic* authors were acquainted. How different is the treatment almost every practitioner employs from that recommended by those writers whom he has consulted, and still continues to consult, in every doubtful case that may occur, and from whom, on most subjects, instruction may be drawn. Allow me to introduce one or two cases in illustration of this remark, and as a foundation for the inferences which will follow.

*July* 10, 1846. Alexander Walker, aged about 35, a farmer. Attacked on the 8th with fever, the excitement rising in the fore part of the day, obtaining its maximum in the latter part, and subsiding at night.

At 7 o'clock, A. M., third day since the attack. Says he has no fever, but has passed a restless night, having slept but little. Pulse slightly accelerated and moderately full. Skin moist. Much headache and pain in the back. Tongue coated, of a yellowish white color, and complains that everything tastes bitter. Sighs frequently. He says he has been freely catharticised.

*Treatment.*—Sulphate of quinia gr. xx, calomel gr. x; to be followed in one hour by quinia gr. x, opium gr. iij, ipecac. gr. j. Cook's pills to be taken at night. Vomited about an hour after taking the second dose, but thinks he did not throw up much of the medicine. He had no pain or other uneasy sensation, but sweated most profusely, though the pulse became considerably increased in frequency and fullness during the period corresponding with the febrile exacerbation. The cathartic operated only slightly.

*July* 11. During the day there was considerable fever, with headache, restlessness, and thirst. Ten grains of quinia were administered in the morning, followed, in the latter part of the day, by tinct. rhubarb and aloes and calomel, and, as this did not operate, castor oil was given at night with the effect of procuring free evacuations.

*July* 12. Ten gr. of quinia were given early in the morning, repeated once in two hours, till three doses were administered. Had no fever, or headache, or other "misery." Pulse slow and full. Bathed in profuse perspiration except the hands and feet, which are dry and warm, while the head and trunk feel decidedly cool to the touch, in consequence, doubtless, of the rapid evaporation from the surface. A bystander was solicitous on account of the coldness, but, on being apprized of the difference between a cool body with warm hands and feet, and the reverse, he seemed satisfied. Eyes dull, and there seems, from the beginning, to have been a congestion of them with the so-called *determination* of blood to the head. Tongue rapidly cleaning.

*July* 13. Has had no more febrile excitement. Tongue entirely clean. Conjunctiva clear. Has lost his solicitude with regard to his own case, and is in bright hope of recovery. No further treatment was necessary. The sedative power of quinia, in full dose, was strikingly manifest in the complete absence of morbid excitement, with a return of the secretories and capillaries to the performance of their proper offices.

*Sept.* 6th, 1845. Alfred Williams, aged about twenty-five, after severe labor and considerable exposure, was attacked with chilly sensations and feverish blushes, with head-ache, pains in the extremities, bitter taste, fevered tongue, and feeling of great prostration.

*Sept.* 7th, 10, A. M. Says his bowels moved yesterday, and have done so regularly without medicine. Pulse 100 and moderately full. Skin dry, though not extremely so. Thirst considerable, and every thing has a bitter taste.

*Treatment.*—Emetic of Ipecacuanha gr. xx, Tartrate of Antimony and Potash gr. iv. This operated freely, bringing away a large quantity of yellow viscid substance, (bile,) and producing two or three stools. Perspiration occurred during emesis, but the febrile dryness very soon returned.

At 7, P. M., directed—

R.—Sulph. Quinia gr. xv,
Acetate of Morphia gr. iss,
Pulv. Doveri gr. xij.

*Sept.* 8th, 7, A. M. Bowels moved once or twice soon after the administration of the above prescription. Slept well during the night, though he frequently waked, and occasionally asked for water. Head-ache much diminished. The dense coating on the tongue seemed somewhat lessened. Pulse 72 and full. Skin a little drier than natural. At the time of taking the above he was slightly delirious, but this morning he is entirely rational. Directed Sulph. Quinia gr. x, Dover's Powder gr. xx.

*Sept.* 8th, 5, P. M. Rested in the morning after taking the medicine, and then got up and walked out of doors, vomited, and during two or three hours after noon had considerable thirst, with an aggravation of his other febrile symptoms. Fever has now somewhat subsided. Pulse 100, moderately full, and less quick than this morning. Tongue more coated, and complains of extremely unpleasant taste. Administered six Cook's pills.

*Sept.* 8th, 9, P. M. Vomited soon after taking the pills, and the stomach remained irritable, while the febrile excitement increased. Administered Ipecac. gr. xx, Tart. Ant. et Potash gr. iv, and directed—

R.—Quinia gr. x,
Opium gr. iij,
Ipecac. gr. ij.

to be taken after the free evacuation of the stomach and bowels.

*Sept.* 9th, 5, A. M. He vomited freely, but was restless till the medicine operated as a cathartic, five or six times, about two o'clock. He took the above at half-past two, after which he rested quietly. Skin rather dry, though moist in places. Tongue more coated than yesterday. Thirst moderate, not much uneasiness of head. Pulse 80, moderately full and not quick. Directed—

R.—Quinia gr. x,
Pulv. Dov. gr. xx,
Acetate of Morphia gr. i.

to be taken at 7 o'clock. At 12 o'clock ten grains of Quinia and twenty of Dovers Powder were given, and at night the dose was repeated, with the addition of ten grains of Calomel, and at two o'clock the last dose was repeated.

*Sept.* 10th. During the day, yesterday, he remained thirsty, though the deeply coated tongue was moist, and there was a slight appearance of *cleaning*. He was much nauseated in the night, and a sinapism was applied to the epigastrium.

*Sept.* 10th, 7, A. M. Gave—

R.—Ext. Colocynth comp. gr. xx,
Sulph. Quinia gr. x.

This produced four or five evacuations about ten o'clock. Took a teaspoonful of Paregoric after the last discharge, which was thin, and at two, P. M. Gave—

R.—Quinia gr. x,
Dov. Pow. gr. xx.

and with the exception of a short period of nausea, with the vomiting of a small quantity of yellowish green, viscid, and bitter substance, (bile,) he passed a very comfortable evening. The skin has not been moist to-day, but has presented a very natural appearance. The coating on the tongue, though dense, and, in the middle, brown, seems to be loosening at the tip and sides. Directed that—

R.—S. Quinia gr. v,
Pulv. Dov. gr. x.

should be given at 9 and repeated at 3 o'clock.

*Sept.* 11th. He vomited the first dose and did not take the second; was much nauseated all night. Pulse 80. Skin not unnatural. Tongue rapidly cleaning, and over the portions denuded of the coating it appears of a brighter red than natural. Says he feels very well. Drank small quantities of brandy during the day. Administered at night Calomel and Rhubarb, of each five grains, to be followed in the morning by Quinia and Dover's Powders, of each ten grains.

*Sept.* 12th. Though the bowels did not move, the Quinia and Dover's Powder were given about three o'clock, and he passed a very comfortable night. Although the general appearance was not materially altered, the thirst had nearly disappeared, and the tongue is more clean.

*Sept.* 15th. From this time he continued to rapidly improve; his appetite soon came, and no more active treatment was resorted to. Brandy and Quinia, in small doses, were occasionally taken as a tonic.

He is to-day walking about. Prescribed a decoction of gentian, cham. flowers, orange peel and serpentaria. He became speedily restored to his usual strength.

Though, in the division of disease previously made, this should be considered a severe case of remitting fever, in its simplest form, yet in the loose nomenclature, which many physicians are in the habit of using, it would probably be called a case of *congestive* fever.

The effect of an active antiperiodic treatment to break in upon the regularity of the remission and exacerbations when it does not fully and at once control the disease, is exemplified in this case, as well as the sedative influence of large doses of Quinia, and the failure of large doses of opium to produce, in the condition of the system existing in many cases of fever, the effects we are accustomed to expect when the remedy is administered in health. A similar modification of effect is also noticed in quinia, for although tinnitus aurium and headache, with a feeling of distension of the eyes are common effects of this remedy, though less so in doses of ten grains than of two, yet while the system is laboring under a *smothered* fever with restlessness, head-ache, pains in the back and extremities, and general torpor of the secretories, we frequently see none of these unpleasant symptoms manifested, but a complete removal of all the unpleasant sensations the patient experienced, before taking the medicine. This happy effect of quinia seems to be favored by combination with a full narcotic dose of opium, or its principal alkli morphia; thus, by combining five, ten, fifteen or twenty grains of sulphate of quinia with from a fourth of a grain to a grain of sulphate or acetate of morphia, we have a combination which acts far more mildly and pleasantly than would either of the remedies singly. Not unfrequently patients who have tossed, and groaned, and fretted for a great part of the time for twenty-four or forty-eight hours, have, in the course of an hour, after taking a full dose of quinia and morphia, passed into a pleasant state of waking dreams, with moist tongue and skin, with soft pulse, and in their elysium have forgotten all their sufferings, though they may have assured their physician that they "never could take quinia or opium without the most disagreeable feelings." As it is not every case which demands the remedy in large doses, it is an important inquiry, what is the sign of its necessity? Certainly not the coldness of the chill, nor the heat of the fever, but the nervous prostration and the disturbance of capillary action, resulting in congestion, showing a powerful influence of malerious poison. This conclusion is supported by two facts, often observed by the practitioner, viz:

1st, The most speedily fatal cases are those in which there is little or no chill, and little or no febrile excitement; and, sometimes on the other hand, a diminution of the frequency of the pulse and impairment of muscular power, with torpor of the secretories, without the dryness which would result from febrile reaction, and often there is a relaxation of the cutaneous exhalents resulting in the cold sweat, so generally accompanying the cadaverous countenance, speaking defiance to science and art. And,

2d, The cases which bear the largest doses with no other apparent influence of the remedy, than a silent but effectual restoration of the system to its proper tone and activity, are precisely those dangerous cases just mentioned. Where there is not a powerful impression of malaria upon the system, and a quick sensibility to impressions remain, the medicine does not often operate noiselessly or always sightlessly to the patient, but sights and sounds follow each other in rapid succession. Indeed chill or fever seems not to be at all essential to a malarious disease, which may pursue its course as regularly as though fever were present, and the writer's own case is in point. After having been exposed to the worst atmospheric influence, presented by the hot season, with irregular sleep, and a deficiency of it, there insidiously crept on, a diminution of appetite, a coating upon the tongue, general soreness of the muscles, and inability to move the head suddenly without pain, which in about two days resulted in vertigo, and such a feeling of prostration, as entirely forbid further exertion. A high degree of nervous excitement occurred, but this was of short duration; the tongue soon became covered with a dense coating, and under the influence of copious drafts of warm drink, a most profuse sour perspiration occurred. The bowels acted tardily under the influence of mercurial cathartics, and they hardly responded at all to any other. At first the pulse was somewhat accelerated, and once or twice afterwards; but generally the heart's action was perfectly natural, and from beginning to end there were no chilly sensations, nor was there any feeling of fever, but what might be attributed to the use of quinia, which produced a most pleasurable excitement, unalloyed with any of those disagreeable associations which usually characterize reality. Sleep was but little disturbed, and for six days there was a perfect indifference to both food and drink, and there existed no other inducement to take the latter than to fill up an uncomfortable void. During this time the tongue was thickly coated, and an extremely unpleasant taste resulted from it, but upon the seventh day from the beginning of apparent disease, a slight appetite appeared, the tongue began to clean, and a rapid restoration followed. Here was a bilious disease, and nothing afforded so much apparent benefit as a dose of thirty grains of calomel, which produced one or two copious bilious stools. Now this certainly was not a bilious fever, or any other fever, according to the common acceptation of that term. Though the symptoms varied, there were no regular periodic exacerbations and remissions, and consequently the same apparant benefit could not be perceived, which unequivocally follows the use of appropriate remedies in periodical affections. The same remedies were however used, and with seeming advantage.

From what has been said, it probably will not be inferred that the writer thinks lightly of preparatory evacuants before giving quinia alone or combined; a remedy which seems at the same time, anti-periodic and anti-malarial; for in the great majority of cases, it seems indispensable that a satisfactory and pleasant effect of the remedy must be preceded by an emetic or cathartic, and where a thick coating has appeared upon the tongue in the early stages of the disease, without evidence of mucous irritation, an emetic of four grains of Tart. Antimony and Potassa, with twenty grains of Ipecacuanha have been preferred to a cathartic, but in all other cases the latter has been thought preferable.

To quiet the irritability of the mucous membrane, with the disposition to flatulence so common in fever, oil of turpentine given in doses of a few drops, or in that of one or two drachms, to quicken the action of other cathartics, has seemed more effectual than any thing else which I have seen employed. Its vapor readily diffuses itself through the whole of the alimentary canal, imparting a healthful tone, and stimulating the dilated capillaries to contract.

To quiet the nausea so apt to follow the taking of quinia, a brisk sinapism usually proves effectual, and in consequence of the pretty uniform occurrence of such a condition, the writer has not latterly been in the habit of giving quinia either alone or combined, in doses nearer together than five or six hours; and Ipecac. he has discontinued as an accompaniment for quinia; for, without seeming to increase the diaphoresis resulting from a full dose of quinia and morphia, it greatly augments the most troublesome nausea, and occasionally results in the loss of ten or fifteen grains of valuable medicine, and so offends the stomach that it will not immediately receive another dose.

At a future time the writer may trouble this association with further remarks upon the effects of sulphate of quinia, upon our fevers, and upon the use of other remedies, which have not here been mentioned.

